# Efficacy and risk factors of stent placement in the treatment of malignant tracheoesophageal fistula

**DOI:** 10.3389/fonc.2024.1421020

**Published:** 2024-08-06

**Authors:** Qingxia Wang, Zhihong Duan, Shiqi Liu, Ruihua Shi

**Affiliations:** ^1^ Department of Gastroenterology, Southeast University Affiliated Zhongda Hospital, Medical School, Nanjing, China; ^2^ Department of Gastroenterology, Bringing Enjoyment and Quality to Life (BENQ) Medical Center, Nanjing, China

**Keywords:** tracheoesophageal fistula, esophageal stent, tracheal stent, conservative treatment, risk factors

## Abstract

**Background:**

Due to the low incidence of malignant tracheoesophageal fistula and the paucity of relevant clinical studies, the benefits of stent implantation have not been well documented. It remains unclear which factors may affect fistula closure.

**Methods:**

Between January 2015 and January 2021, 344 patients who were diagnosed with malignant tracheoesophageal fistula at Zhongda Hospital, Southeast University, were retrospectively enrolled. Demographic and clinical data were collected. Risk factors for fistula closure identified by univariate analysis were further analyzed using multivariable logistic regression.

**Results:**

A total of 288 patients were analyzed in this study, of which 94 were treated conservatively, 170 were treated with an esophageal stent, and 24 were treated with a tracheal stent. Among them, the delta Karnofsky’s performance status score values (after 2 weeks/before treatment [p = 0.0028], after 1 month/before treatment [p = 0.0103]) were significantly different between conservative and stent treatment. There was a significant reduction of pneumonia incidence in the stenting group (33.53%) compared to the conservative treatment group (77.05%) after one month (p <0.0001). In addition, the closure of fistulas was influenced by four independent risk factors: 1) treatment methods (p < 0.0001), 2) fistula size (p = 0.0003), 3) preoperative white blood cell count (p = 0.0042), and 4) preoperative Karnofsky’s performance status score (p = 0.0001).

**Conclusions:**

Stent implantation has become an effective method for treating malignant tracheoesophageal fistula compared to conservative treatment. Additionally, stent implantation, smaller fistula size, lower preoperative white blood cell count, and higher preoperative Karnofsky’s performance status score suggest a better outcome.

## 1 Introduction

Malignant tracheoesophageal fistula (mTEF) is a clinically refractory disease mainly caused by esophageal and bronchial cancers (1, 2). A variety of complications (e.g., malnutrition, lung infection) caused by mTEF may lead to deterioration of the patient’s condition and even death from respiratory failure (3, 4). Recent studies suggest that mTEF is often associated with decreased survival time, ranging from 1 week to 12 months (1, 5). Currently, the goals of treatment are to seal the fistula and improve the quality of life.

The predominant modalities to seal the fistula include conservative treatment and endoscopic stent implantation (6, 7). Conservative treatment (e.g., gastrostomy, nasogastric tube, and antibiotics) has been the most common treatment for patients with mTEF, primarily to improve their nutritional status and to alleviate lung infections (8). The efficacy of conservative treatment, however, is limited. The endoscopic stent implantation technique has revolutionized the therapeutic prospects of mTEF (9, 10). Stent implantation can effectively increase the rate of fistula closure by physically occluding it (5, 11). Some parameters may influence the closure of fistula, such as fistula location (9) and preoperative chemoradiotherapy (12). Nevertheless, due to the low incidence of mTEF (1, 7, 13) and the paucity of relevant clinical studies, the benefits of stent implantation have not been well documented. It remains unclear which factors may affect fistula closure.

Thus, the aim of this study is to evaluate the efficacy of stent implantation in the treatment of mTEF and to identify risk factors that may have an impact on fistula closure in mTEF.

## 2 Materials and methods

### 2.1 Study cohort

**Figure 1:**
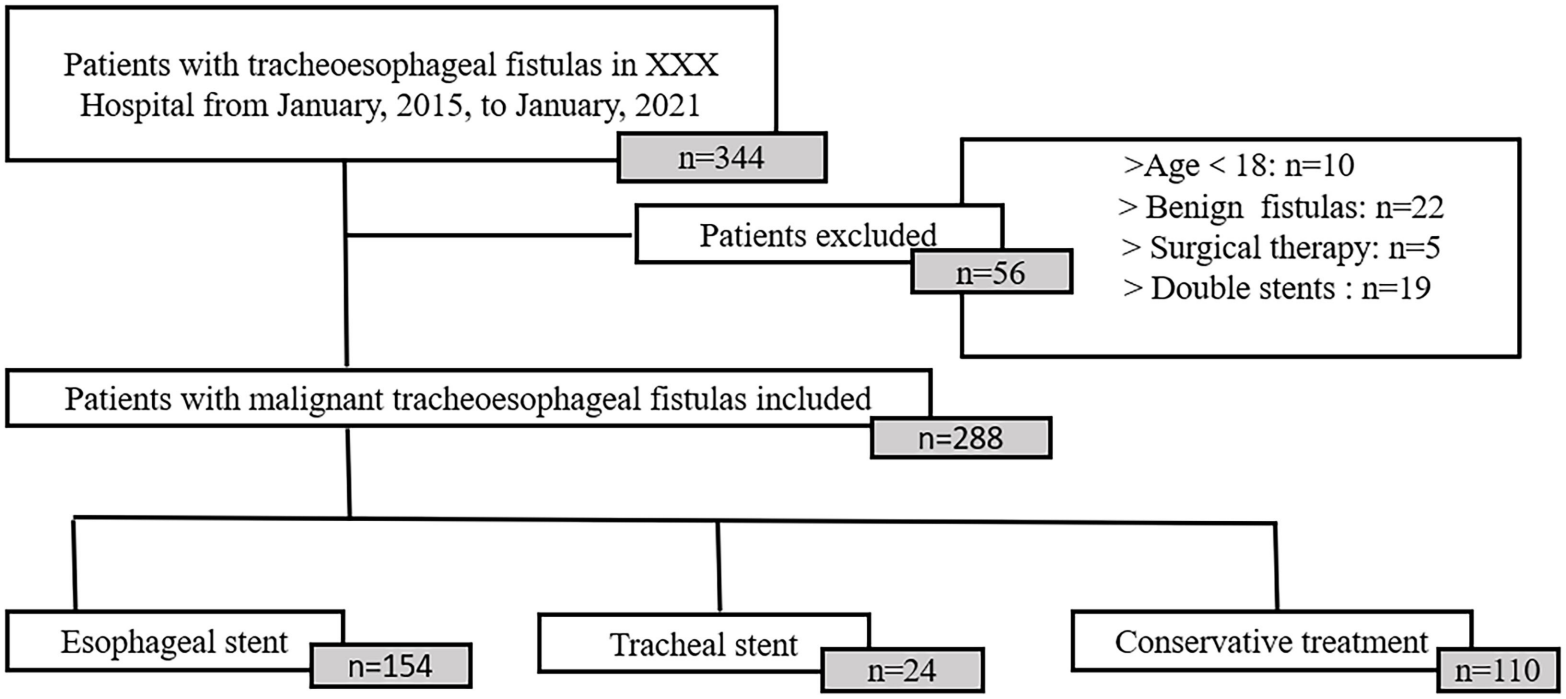
Flow chart of patient selection.

The study was designed as a retrospective cohort study, including 344 patients with mTEF retrieved from Zhongda Hospital between 2015 and 2021. The diagnosis of mTEF was based on a combination of clinical symptoms, pathology, radiologic (**Supplementary Figures 1**, **2**), and endoscopic evidence (**Supplementary Figure 3**) of transesophageal fistulas. All patients treated with conservative therapy or stent therapy were included. Conservative therapy mainly consisted of antibiotics, gastric tube or intestinal tube feeding, and gastrostomy or jejunostomy. Stent therapy included esophageal stent placement or tracheal stent placement (**Supplementary Figure 4**). Only self-expanding metal stents with silicone membranes were used in the esophagus. Skirted and wired esophageal stents are often used to prevent migration. Straight and Y-shaped silicone stents were used in trachea. All stents should extend 2 cm beyond the upper and lower margins of the fistula. The choice of treatment mainly depended on the location and size of the fistula and whether the airway was compressed. The clinical data of 288 patients who met the inclusion criteria were finally obtained (**Figure 1**). This study was approved by the IEC for Clinical Research of Zhongda Hospital, affiliated with Southeast University, in conformity to Helsinki Protocol.

### 2.2 Data collection

The clinical data (e.g., gender, age, tumor indexes, fistula location, fistula size, laboratory indexes, and previous medical history) were collected from the electronic medical records before the operation using the Hospital Information System (Neusoft Group Co., Ltd.) and Laboratory Information System (Neusoft Group Co., Ltd.). Karnofsky’s performance status score (13) (KPS) is commonly used internationally, splitting patient activity into 11 levels per percent. We assessed KPS scores at patient admission, and then at 2 weeks, 1 month, 3 months, and 6 months after treatment by having patients fill out questionnaires. Pneumonia was determined based on the Clinical Pulmonary Infection Score (14) (CPIS), especially elevated leukocytes and infiltrated on CXR. The documented size of the fistula was measured endoscopically, by close apposition of an endoscopic forceps of a determined size. The criteria for fistula closure were endoscopic evidence of successful fistula closure and upper gastrointestinal imaging evidence of no leakage of contrast medium. Data on adverse reactions were subsequently collected. We continue to follow the patient until the patient dies, or the patient is lost to follow-up.

### 2.3 Statistical analysis

JMP Pro 15 (SAS Institute Inc., NC, USA) was applied for statistical analysis in this study. Continuous variables and categorical variables were reported as mean (± SD) and counts (percentage), respectively. The qualitative data were compared using the Chi-square test or Fisher exact test, as appropriate. Comparison of quantitative data between two and multiple groups was performed by t-test and analysis of variance (ANOVA), respectively. Logistic regression analysis was applied to assess the independent risk factors for fistula closure. A two-sided p-value <0.05 was considered statistically significant.

## 3 Results

### 3.1 Patient characteristics

A total of 288 patients met the inclusion criteria, of which 94 (32.64%) were treated conservatively, 170 (59.03%) were treated with esophageal stent therapy, and 24 (8.33%) were treated with tracheal stent therapy. There were 208 (72.22%) males and 80 (27.78%) females. The mean age of all patients was 63. 85 ± 10. 52 years old. A total of 258 patients suffered tracheoesophageal fistula due to esophageal cancer, while 21 (7.29%) patients encountered tracheoesophageal fistula due to pulmonary cancer. Regarding the location of the fistula, 134 cases (46.53%) were in the upper esophagus, 136 cases (47.22%) in the middle esophagus, and 18 (6.25%) cases in the lower esophagus. The average diameter of the fistula was 2. 09 (± 0. 69) cm. The mean preoperative leukocyte and platelet counts were 8.55 (± 4.24) × 10^9^/L and 256.10 (± 111.54) × 10^9^/L. The average body mass index was 18.83 ± 2.86. The mean preoperative KPS was 49.48 ± 10.21 (for patient characteristics see **Table 1**).

**Table 1:** Demographics and characteristics of patients included in the study.

Characteristics	Total N (%)
**Age, *mean ± SD, years***	63.85 ± 10.52
Gender	
Male	208 (72.22)
Female	80 (27.78)
Smoke	
Yes	80 (27.78)
No	208 (72.22)
Alcohol use	
Yes	52 (18.06)
No	236 (81.94)
Tumor type	
Esophageal cancer	258 (89.58)
Pulmonary cancer	21 (7.29)
Other tumors	9 (3.13)
Tumor stage	
T1	49 (17.01)
T2	153 (53.12)
T3	52 (18.06)
T4	34 (11.81)
Lymph node metastasis	
Yes	186 (64.58)
No	102 (35.42)
Metastasis	
Yes	53 (18.40)
No	235 (81.60)
Pneumonia	
Yes	235 (81.60)
No	53 (18.40)
Fistula location	
Upper esophagus	134 (46.53)
Middle esophagus	136 (47.22)
Lower esophagus	18 (6.25)
**Fistula size (cm)**	2.09 ± 0.69
Previous therapy	
Previous interventional therapy	125 (43.40)
Previous surgical treatment	135 (46.88)
Previous radiotherapy	144 (50.00)
Previous chemotherapy	146 (50.69)
Treatment	
Conservative treatment	94 (32.64)
Esophageal stent	170 (59.03)
Tracheal stent	24 (8.33)
Preoperative indexes	
CEA	5.16 ± 16.87
CA-199	14.35 ± 15.53
NSE	13.10 ± 10.27
CK19	5.05 ± 7.33
Pre-WBC	8.55 ± 4.24
Pre-PLT	256.10 ± 111.54
Pre-N%	79.07 ± 10.96
INR	1.23 ± 0.16
D-dimer	549.35 ± 771.20
BMI	18.83 ± 2.86
Pre-albumin	35.30 ± 4.62
Pre-KPS	49.66 ± 10.33

Unless otherwise indicated, date are numbers of participants, with percentages in parentheses. WBC, white blood cell count; Pre-N%, preoperative Neutrophil ratio; CEA, Carcinoembryonic antigen; CA-199, Carbohydrate antigen-199; NSE, Neuro-specific enolase; CK19, Cytokeratin 19 fragment; PLT, platelet; INR, international normalized ratio; BMI, body mass index; Pre-albumin, preoperative albumin.

### 3.2 Efficacy of treatments

To clarify the efficacy of different treatments (i.e., conservative, esophageal stent, and tracheal stent treatment), we assessed the patients’ KPS scores at different time points before treatment and at 2 weeks, 1 month, 3 months, and 6 months after treatment. The differences (delta) in KPS scores before and after treatments were calculated and compared. The delta KPS values (after 2 weeks/before treatment [p = 0.0028], after 1 month/before treatment [p = 0.0103]) were significantly different between conservative and stent treatments (**Table 2**). However, we did not find the difference between esophageal stent and tracheal stent treatments (**Table 3**).

**Table 2:** KPS scores of patients in the conservative and stent groups.

	Conservative therapy (n)	Stent therapy (n)	P-value
Preoperative KPS score	45.11 ± 1.02 (94)	51.80 ± 0.71 (194)	<0.0001
d-value of 2 weeks	3.75 ± 0.74 (92)	6.47 ± 0.51 (194)	0.0028
d-value of 1 month	5.49 ± 1.33 (61)	9.48 ± 0.79 (173)	0.0103
d-value of 3 months	7.50 ± 1.82 (44)	9.50 ± 1.06 (130)	0.3445
d-value of 6 months	7.00 ± 2.49 (35)	6.46 ± 1.66 (79)	0.8558

d-value means difference between postoperative KPS score and preoperative KPS score.

**Table 3:** KPS scores of patients in esophageal and tracheal stent group.

	Esophageal stent (n)	Tracheal stent (n)	P-value
Preoperative KPS score	52.00 ± 0.68 (170)	50.42 ± 1.81 (24)	0.4142
d-value of 2 weeks	6.44 ± 0.49 (170)	6.67 ± 1.30 (24)	0.8713
d-value of 1 month	9.31 ± 0.77 (152)	10.71 ± 2.07 (21)	0.5252
d-value of 3 months	8.82 ± 1.06 (114)	14.38 ± 2.82 (16)	0.0670
d-value of 6 months	5.29 ± 1.83 (69)	14.50 ± 4.80 (10)	0.0771

d-value means Difference between postoperative KPS score and preoperative KPS score.

Pneumonia was also evaluated to assess the effect of conservative treatment and stent implantation. Before treatment, there was no significant difference (p = 0.2357) in the occurrence of pneumonia between the conservative treatment and stenting groups, with 73 cases (77.66%) in the conservative treatment group and 162 cases (83.51%) in stenting group. After one month of treatment, there was a significant reduction in the incidence of pneumonia (33.53%) in the stenting group, while 77.05% of the patients in the conservative treatment group still had pneumonia (p <0.0001). The results showed that infection control in the stent group was significantly better than in the conservative treatment group (**Table 4**).

**Table 4:** Pneumonia of patients in the conservative and stent groups.

	Conservative therapy	Stent therapy	P-value
Preoperative pneumonia	73/94(77.66%)	162/194(83.51%)	0.2357
1 month pneumonia	47/61(77.05%)	58/173(33.53%)	<0.0001

### 3.3 Risk factors

We performed the univariate and multivariable analyses of 288 patients to identify the independent risk factors of fistula closure. In the univariate analysis, the data showed that age (p = 0.0117), body mass index (p = 0.0456), metastasis (p = 0.03), one month pneumonia (p <0.0001), treatment (p <0.0001), fistula size (p <0.0001), fistula location (p = 0.0399), preoperative white blood cell count (pre-WBC) (p <0.0001), preoperative neutrophil ratio (pre-N%) (p = 0.0235), and preoperative-KPS (pre-KPS) (p <0.0001), had a significant impact on fistula closure. In the further multivariable analysis, we found that pre-WBC (p = 0.0042), pre-KPS (p = 0.0001), fistula size (p <0.0003), and treatment (p <0.0001) were statistically significant independent predictors of fistula closure (**Table 5**).

**Table 5:** Univariate and multivariate regression of risk factors of fistula closure.

Variables	Univariate Regression			Multivariate Regression	
	Non-closure (n = 89)	Closure (n = 189)	P-value	OR (95%CI)	P-value
**Age**	66.17 ± 1.10	62.77 ± 0.76	0.0117	1.02 (0.96 to 1.09)	0.5273
**Previous chemotherapy**			0.2110		
Yes	39 (43.82%)	98 (51.85%)			
No	50 (56.18%)	91 (48.15%)			
**Previous radiotherapy**			0.1537		
Yes	38 (42.70%)	98 (51.85%)			
No	51 (57.30%)	91 (48.15%)			
BMI	18.32 ± 0.30	19.06 ± 0.21	0.0456	1.02 (0.83 to 1.26)	0.8452
**Tumor stage**			0.2942		
T1	16 (17.98%)	33 (17.46%)			
T2	43 (48.31%)	105 (55.56%)			
T3	15 (16.85%)	34 (17.99%)			
T4	15 (16.85%)	17 (8.99%)			
**Lymph node metastasis**			0.7209		
Yes	58 (65.17%)	119 (62.96%)			
No	31 (34.83%)	70 (37.04%)			
**Metastasis**			0.03		0.6523
Yes	23 (25.84%)	28 (14.81%)		1.38 (0.34 to 5.67)	
No	66 (74.16%)	161 (85.19%)		reference	
**Pre-pneumonia**			0.3261		
Yes	69 (77.53%)	156 (82.54%)			
No	20 (22.47%)	33 (17.46%)			
**1 month-pneumonia**			<.0001		0.0470
Yes	45 (75.00%)	59 (34.30%)		3.50 (1.02 to 12.03)	
No	15 (25.00%)	113 (65.70%)		reference	
**Treatment**			<.0001		<.0001
Conservative treatment	78 (87.64%)	13 (6.88%)		reference	
Esophageal stent	10 (11.24%)	156 (82.54%)		0.01 (0.00 to 0.05)	<.0001
Tracheal stent	1 (1.12%)	20 (10.58%)		0.00 (0.00 to 0.07)	0.0002
**Fistula Size*(centimeter)***	2.40 ± 0.07	1.98 ± 0.05	<.0001	8.94 (2.71 to 29.48)	0.0003
**Fistula location**			0.0399		0.4938
Upper	48 (53.93%)	80 (42.33%)		reference	
Middle	39 (43.82%)	93 (49.21%)		0.60 (0.17 to 2.22)	0.6042
Lower	2 (2.25%)	16 (8.47%)		0.27 (0.02 to 3.23)	0.2651
**Pre-WBC**	10.30 ± 0.44	7.67 ± 0.30	<.0001	1.25 (1.07 to 1.45)	0.0042
**Pre-N%**	81.10 ± 1.17	77.87 ± 0.80	0.0235	0.99 (0.92 to 1.08)	0.9512
**Pre-KPS**	44.61 ± 0.98	52.01 ± 0.67	<.0001	0.88 (0.82 to 0.94)	0.0001
**Pre-albumin**	35.11 ± 0.50	35.46 ± 0.35	0.5587		

CI, confidence interval; OR, odds ratio.

### 3.4 Complications

The therapeutic safety of stent implantation was evaluated in terms of complications. There were different kinds of complications after stent implantation.

In the esophageal stent group, 69 patients had early (≤ 24 h) complications, including 49 patients with retrosternal pain (28.82%), seven patients with asthma (4.12%), six patients with hematemesis (3.53%), five patients with stent displacement (2.94%), and two patients with tracheal compression (1.18%). Eleven patients had late (≤24 h) complications, including six patients with retrosternal pain (3.53%), two patients with dyspnea (1.18%), one patient with stent obstruction (0.59%), one patient with hematemesis (0.59%), and one patient with vomiting (0.59%). When patients experienced a stent-related complication (such as obstruction, hematemesis, stent displacement), there should be a low threshold to re-image and perform endoscopy, which is not unusual.

In the tracheal stent group, nine patients had early (≤ 24 h) complications, including six with retrosternal pain (25.00%), one with hemoptysis (4.17%), one with stent displacement (4.17%), and one with asphyxia (4.17%). Only two patients had long-term (>24 h) complications in the tracheal stent group: one had retrosternal pain (4.17%) and one had asphyxia (4.17%). Once a patient develops stent displacement or asphyxia, endoscopic reintervention should be considered, such as immediately stent removal or intubation.

## 4 Discussion

Malignant TEF is considered a devastating disease, with a life expectancy of only a few months (4). The incidence of mTEF is 5%–15% (1, 3). The occurrence of mTEF is mainly due to esophageal carcinoma, while less than 10% of mTEF is caused by pulmonary carcinoma (1, 3, 7, 15). Our data suggested that 89.58% of patients with mTEF were caused by esophageal carcinoma and 7.29% by lung carcinoma, which was consistent with the results of earlier studies. Of note, due to the low incidence of mTEF and the short survival period, most previous studies consisted of fewer than 100 patients (7, 15–17). In our study, we successfully analyzed 288 patients to compare the efficacy of conservative and stent implantation treatments and to identify the independent factors that impact fistula closure in mTEF.

Since a number of studies have reported that stent implantation could seal fistula defects and improve symptoms of pneumonia, endoscopic stent implantation is recommended for the treatment of mTEF, whereas surgical treatment approaches can only be considered in individual cases or specialized clinical center (4, 18). To the best of our knowledge, the benefit of endoscopic stent implantation has not been well documented. Our data indicated that stent implantation effectively improved KPS scores and controlled lung infections with a high fistula closure rate (176/187, 94.12%), but no difference was found between esophageal and tracheal stents. This was consistent with some studies which suggested that no specific type of stent had obvious advantages (15, 19). Esophageal stents and tracheal stents had very high fistula closure rates (67%–100% (15, 16), regardless of shape, material, etc. Moreover, although double stenting is reserved for a minority of cases, and studies on the efficacy or safety compared to standalone esophageal or airway stenting are lacking, some case series suggest that double stenting may improve survival (9). Furthermore, some experts suggest that double stenting should be considered in large fistula (> 2 cm) or in patients with airway compression after esophageal stent placement. Some new techniques, such as fascia lata graft placement between two stents, might represent a viable option for more complex cases (20). These treatments can also provide ideas for subsequent research.

To further evaluate the factors that may influence fistula sealing, univariate and multivariable analyses were performed. We found that pre-WBC, pre-KPS, fistula size, and treatment modalities were statistically significant independent factors of fistula closure. The pre-WBC reflected the degree of inflammation reaction, which possibly caused tissue edema and affected tissue repair. The pre-KPS was a health care provider–administered assessment that reflected the patient’s ability to take care of himself and tolerate the side effects of treatments. Large fistula size was associated with unsuccessful closure in three studies that used a cutoff of 1.5 cm, 2.0 cm, and 3.0 cm (21–23). Our data also concluded that the larger the fistula, the more difficult it is to close. Furthermore, some investigators found that etiology, fistula location, fistula size, time from diagnosis to stent placement, and inflammation degree may affect the fistula closure (9, 19, 21–24). However, we did not find any effect of previous radiotherapy or chemotherapy on fistula sealing. Some studies (12, 25, 26) showed that previous radiotherapy and chemotherapy hampered the healing of the fistula and affected the prognosis of mTEF. This discrepancy may be due to the fact that conventional radiotherapy and chemotherapy usually affected a much larger area than where the tumor was located, causing significant damage to normal tissues. With advances in radiotherapy, perioperative brachytherapy can now achieve higher accuracy in positioning and steeper dose decay outside the target area, thereby reducing the extent of normal tissue damage around the fistula (27).

Despite careful stent placement techniques, stent-related complications cannot be completely avoided because the stent is a foreign body that can promote granulation tissue proliferation in the esophagus (28). We can improve the efficacy of stent therapy and reduce the incidence of stent-related complications by enhancing stent configuration and materials (29). The main limitations of our study are its retrospective design and single-center scope. The choice of treatment was ultimately decided by patients, which led to a risk of selection bias. Additionally, this single tertiary center study may suffer from referral bias. It would have been ideal, but not practical, to have a prospective randomized comparison group study of conservative treatment versus stenting. Secondly, it is not clear whether the higher preoperative white blood cells in the non-closure group reflected inflammation at the fistula site or pulmonary inflammation. Thirdly, the documented size of the fistula depended on the subjective evaluation by the performing endoscopist, and the recorded KPS scores were based on subjective perceptions by patients. Moreover, our team attempted to perform a survival analysis, but due to the severity of mTEF itself, very few patients survive more than 6 months postoperatively, making the survival analysis curves un convincing (**Supplementary Figure 5**). Certainly, further prospective studies with larger sample sizes and/or multicenter studies are needed to validate the findings of this study.

In conclusion, stent implantation is an effective treatment for mTEF compared to conservative treatment. Additionally, pre-WBC, pre-KPS, fistula size, and treatment modalities can independently affect the prognosis of fistula sealing in mTEF, which can support physicians in clinical decision-making.

## Data availability statement

The raw data supporting the conclusions of this article will be made available by the authors, without undue reservation.

## Ethics statement

The studies involving humans were approved by IEC for Clinical Research of Zhongda Hospital, Affiliated to Southeast University. The studies were conducted in accordance with the local legislation and institutional requirements. The participants provided their written informed consent to participate in this study.

## Author contributions

QW: Conceptualization, Formal analysis, Methodology, Software, Writing – original draft. ZD: Data curation, Supervision, Writing – review & editing. SL: Data curation, Supervision, Writing – review & editing. RS: Resources, Supervision, Writing – review & editing, Project administration.

## Supplementary material

Supplementary Figure 1
**(A)** The red arrow pointing to the fistula and the presence of contrast leakage; **(B)** the red arrow pointing to the successful closure of the fistula after stenting and the absence of contrast leakage.

Supplementary Figure 2
**(A)** showing a penetrate through the esophagus in mediastinal window; **(B)** showing a penetrate through the esophagus in lung window.

Supplementary Figure 3 pictures of tracheoesophageal fistula under esophagoscopy **(A)** and tracheoscopy **(B)** (red arrows indicate the fistula sites).

Supplementary Figure 4 The esophageal stent **(A)** and the tracheal stent **(B)** blocking the fistula through esophagoscopy and tracheoscopy.

Supplementary Figure 5 The survival curves that we have previously analyzed for your reference (the horizontal coordinate is the survival time in days; the p-values for plots **A**, **B**. are 0.01 and 0.03 respectively).
