# Supplementary figures and images for: Efficacy and risk factors of stent placement in the treatment of malignant tracheoesophageal fistula
Source: Front Oncol. 2024 Aug 6;14:1421020. doi: 10.3389/fonc.2024.1421020 (PMC11333233; doi:10.3389/fonc.2024.1421020)

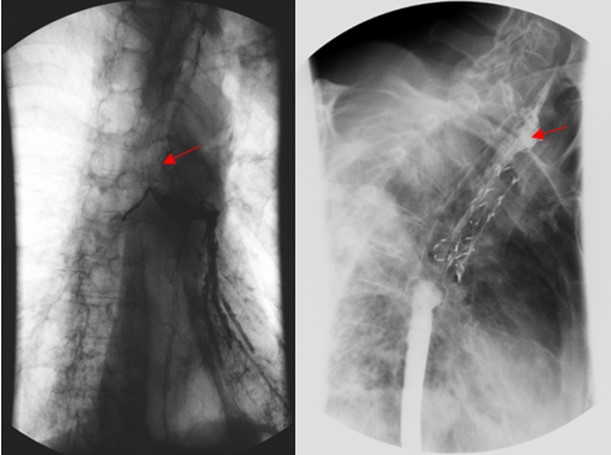

Supplement: Supplementary Figure 1 — (A) The red arrow pointing to the fistula and the presence of contrast leakage; (B) the red arrow pointing to the successful closure of the fistula after stenting and the absence of contrast leakage. [file Image_1.jpg]

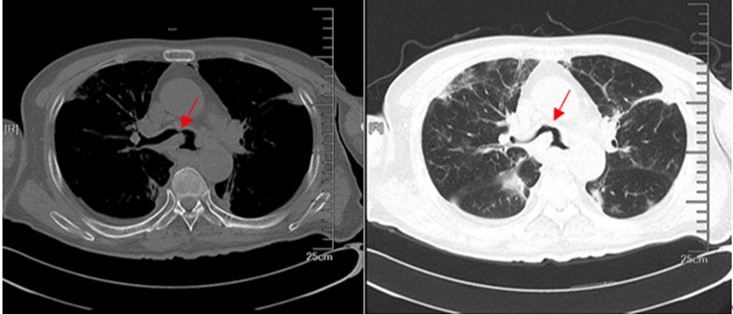

Supplement: Supplementary Figure 2 — (A) showing a penetrate through the esophagus in mediastinal window; (B) showing a penetrate through the esophagus in lung window. [file Image_2.jpg]

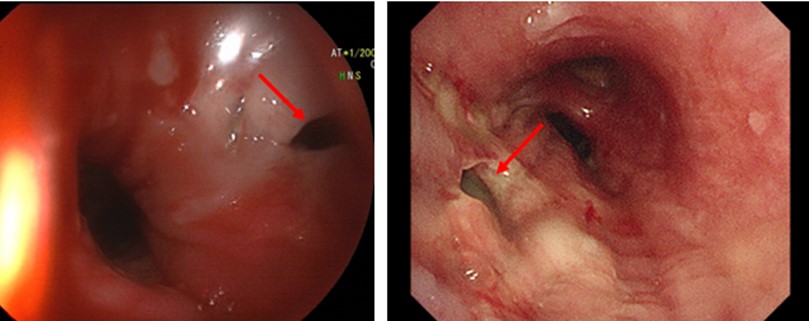

Supplement: Supplementary Figure 3 — pictures of tracheoesophageal fistula under esophagoscopy (A) and tracheoscopy (B) (red arrows indicate the fistula sites). [file Image_3.jpg]

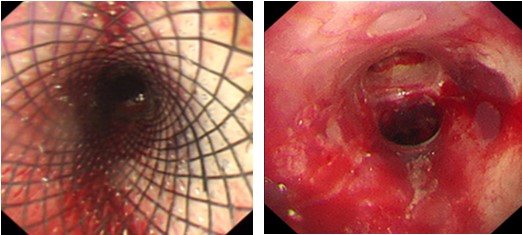

Supplement: Supplementary Figure 4 — The esophageal stent (A) and the tracheal stent (B) blocking the fistula through esophagoscopy and tracheoscopy. [file Image_4.jpg]

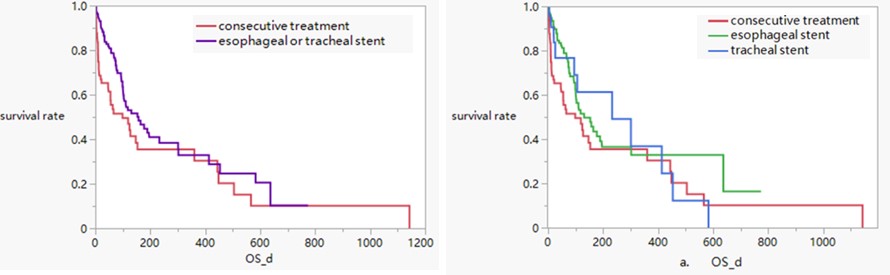

Supplement: Supplementary Figure 5 — The survival curves that we have previously analyzed for your reference (the horizontal coordinate is the survival time in days; the p-values for plots A, B. are 0.01 and 0.03 respectively). [file Image_5.jpg]
